# Improving the cotton simulation model, GOSSYM, for soil, photosynthesis, and transpiration processes

**DOI:** 10.1038/s41598-023-34378-3

**Published:** 2023-05-05

**Authors:** Sahila Beegum, Dennis Timlin, Kambham Raja Reddy, Vangimalla Reddy, Wenguang Sun, Zhuangji Wang, David Fleisher, Chittaranjan Ray

**Affiliations:** 1 Adaptive Cropping System Laboratory, USDA-ARS, Beltsville, MD 20705 USA; 2 Nebraska Water Center, Robert B. Daugherty Water for Food Global Institute, 2021 Transformation Drive, University of Nebraska, Lincoln, NE 68588 USA; 3 Department of Plant and Soil Sciences, Mississippi State University, Mississippi, MS 39762 USA; 4 Department of Plant Science and Landscape Architecture, University of Maryland, College Park, MD 20742 USA

**Keywords:** Light responses, Photosynthesis, Plant development, Plant physiology, Software, Climate-change impacts, Environmental impact

## Abstract

GOSSYM, a mechanistic, process-level cotton crop simulation model, has a two-dimensional (2D) gridded soil model called Rhizos that simulates the below-ground processes daily. Water movement is based on gradients of water content and not hydraulic heads. In GOSSYM, photosynthesis is calculated using a daily empirical light response function that requires calibration for response to elevated carbon dioxide (CO_2_). This report discusses improvements made to the GOSSYM model for soil, photosynthesis, and transpiration processes. GOSSYM’s predictions of below-ground processes using Rhizos are improved by replacing it with 2DSOIL, a mechanistic 2D finite element soil process model. The photosynthesis and transpiration model in GOSSYM is replaced with a Farquhar biochemical model and Ball-Berry leaf energy balance model. The newly developed model (modified GOSSYM) is evaluated using field-scale and experimental data from SPAR (soil–plant–atmosphere-research) chambers. Modified GOSSYM better predicted net photosynthesis (root mean square error (RMSE) 25.5 versus 45.2 g CO_2_ m^−2^ day^−1^; index of agreement (IA) 0.89 versus 0.76) and transpiration (RMSE 3.3 versus 13.7 L m^−2^ day^−1^; IA 0.92 versus 0.14) and improved the yield prediction by 6.0%. Modified GOSSYM improved the simulation of soil, photosynthesis, and transpiration processes, thereby improving the predictive ability of cotton crop growth and development.

## Introduction

The development of cotton crop simulation models started in the mid-1970s with the formulation of fundamental equations for describing growth and development^1–3^. Ritchie^4^ developed the equations for evapotranspiration and soil water balance, and Jones et al.^2^ developed the concept of cotton plant nitrogen (N) balance^2,4^. Modeling cotton fruit development, including squares, bolls, seeds, and fiber, was incorporated in 1988^5^. Since 1983, several models have been developed based on these equations and related theories; GOSSYM^6^, OZCOT^7,8^, CSM-CROPGRO-Cotton^9^, COTCO2 ^10^, and Cotton2K^11^. Among the existing cotton simulation models, GOSSYM has been widely validated and applied in on-farm decision-making and management practices^12,13^. It is a mechanistic, process-level simulation model that estimates crop growth and development based on environmental conditions (solar radiation, temperature, humidity, rain, wind, etc.), soil properties, and management practices (irrigation, fertilizer application)^1,6,14^. Over the years, this model has undergone several improvements and modifications based on advanced concepts and knowledge gained from laboratory, field-scale, and controlled environment experiments^13,13,15–17^. GOSSYM calculates the potential growth of organs as a function of air temperature under optimum water and nutrient conditions^13^. The actual growth rate is determined by reducing the potential growth rate based on water, nutrient, carbon, and temperature stresses^18,19^. GOSSYM runs on a daily time step and provides information about plant size, growth stages, growth rate, various stresses, yield, etc., to the user. The development and applications of GOSSYM are described in several articles^6,13,15^.

### Modeling of photosynthesis and transpiration process in GOSSYM

The primary process in any crop simulation model is the estimation of light interception and photosynthesis. Most crop models (e.g., STICS^20^, APSIM^21^, and CropSyst^22^) estimate crop growth rate using radiation use efficiency- (RUE) based approach in place of photosynthesis^23^. These models are relatively simple in their representation. In most cases, factors that control photosynthesis, such as leaf N content, carbon dioxide (CO_2_) concentration, water stress, nutrient stress, and temperature, are not mechanistically accounted for^24,25^. Comprehensive models with process-level leaf and canopy gas exchange response that can consider the (a) diurnal effect of light, (b) temperature, (c) solar radiation, (d) CO_2_ concentration, (e) soil water status, (f) leaf water potential, (g) relative humidity (h) stomatal conductance and other variables have been developed^26,27^. In GOSSYM, the empirical relationship between light intensity and gross photosynthesis is used for estimating photosynthesis^6^. Potential gross photosynthesis at the maximum solar radiation levels under optimum environmental conditions is determined as a function of incident solar radiation. Gross photosynthesis is then expressed as a product of potential gross photosynthesis per area of the canopy, percentage of light interception, ground area per plant, and a correction factor based on the leaf water potential, leaf N concentration and atmospheric CO_2_ concentration. This is done by considering the entire plant canopy as a photosynthesis element. Net photosynthesis is determined by subtracting maintenance and growth respiration from gross photosynthesis. The calculated net photosynthesis is then distributed as a daily increment of carbohydrates among different plant organs^16^. This method of photosynthesis estimation is relatively simple in its representation. However, there are comprehensive models with process-level leaf and canopy gas exchange response that can consider the (a) diurnal effect of light, (b) temperature, (c) solar radiation, (d) CO_2_ concentration, (e) soil water status, (f) leaf water potential, (g) relative humidity, (h) stomatal conductance and other variables^26,27^. Evapotranspiration in GOSSYM is determined using the evapotranspiration model developed by Ritchie^4^. Transpiration is calculated as a function of leaf area index (LAI) and evaporative demand. The model uses minimum air temperature as the dew point temperature, which will function well in humid conditions but not semi-arid conditions^17^. Evapotranspiration in GOSSYM does not account for the stomatal conductance or atmospheric CO_2_ concentration.

Soil processes in the GOSSYM are simulated by a two-dimensional Rhizos model^6,28^. The model simulates processes in the rhizosphere, including root growth, water flow, nutrient uptake, and transformation. In Rhizos, a slab of soil perpendicular to the row is considered, extending from the row's center to the center of the adjacent row. It considers a two-dimensional geometry in which the size/number of cells is fixed. The slab is represented as a matrix of cells divided into 40 layers, each 5 cm deep, and 20 columns with a width equal to row spacing divided by 20. Each cell assumes similar soil hydraulic properties and solute transport properties. The Gardner- Mayhugh diffusivity function^29^, a simplified Richard's equation based on soil water gradients, and the Marani equation for soil water content release curve^30^ are used for the water flow simulation in the Rhizos model^31^. Water flow simulation in the domain is based on the gradients in the water content in the cells. However, this equation leads to error when the adjacent cells contain soils of different properties. The model is found to give errors in sandy soils because of the model's difficulty in calculating the slope in the diffusivity term^30^. In certain conditions, the water flow calculation based on the water content gradient moves the water flux against the energy potential gradient^30^. In GOSSYM, N can be organic N, ammonium N, and nitrate N. The transformation of organic matter into ammonia and ammonia into nitrate is calculated daily as a function of temperature and soil water content. The model does not consider mineralization from the litter or humus pool and denitrification. Although the current version of the GOSSYM model provides a useful tool for simulating cotton growth and development, it has limitations in the simulation of photosynthesis, transpiration, and soil processes. Therefore, there is a scope for improving the current version of GOSSYM by incorporating more advanced representations of these processes. Such improvements could lead to more accurate and reliable simulations of cotton growth and yield under various environmental and management conditions.

### Improving photosynthesis, transpiration, and below-ground processes in GOSSYM

In plants, photosynthesis and transpiration occur through the stomatal openings. Stomatal conductance is a function of solar radiation, temperature, vapor pressure deficit, soil water status, leaf water potential, CO_2_ levels, and wind velocity. Incorporating stomatal conductance-based estimation of photosynthesis and transpiration has been shown to improve crop growth prediction in different environmental and management conditions^27,32,33^. There have been several studies on developing a mechanistic model for photosynthesis to account for the underlying processes^27,34,35^. The biochemical model for photosynthesis by Farquhar^26^, the stomatal conductance model developed by Ball, Woodrow, and Berry (BWB)^36^, and the leaf-level energy balance model^37,38^ proposed by Kim and Lieth^27^ have been used together for comprehensive modeling of photosynthesis and transpiration in some existing crop models^27,32,39^.

A potential model that can be used to improve the below-ground processes in GOSSYM is the 2DSOIL model. 2DSOIL is a modular, comprehensive two-dimensional soil simulator combined with existing plant models^40^. It is a 2D-finite element-based model that can simulate water flow, solute and heat transport, root growth, and root water uptake in a 2D soil profile^41,42^. Some existing crop models are currently integrated with 2DSOIL to simulate soil processes: SPUDSIM for potato^32^, MAIZSIM for corn^39^, and GLYCIM for soybean^43^. These models provide a soil–plant-atmosphere continuum capable of simulating soil water, crop growth and development, transpiration, and photosynthesis under different environmental and management conditions. In 2DSOIL, water flow simulation is governed by the Richards equation for 2D water flow in variably saturated soil. Solute and heat transport are based on the convective dispersive equation for 2D transport^44^. Root water uptake is a function of leaf water potential, soil water potential, and root and soil resistance. N transformation in 2DSOIL considers the mineralization and immobilization of organic matter, including nitrification and denitrification^45^. Root growth and distribution are based on a convective diffusive 2D root growth and distribution model^42^. It also has a convective diffusive root N uptake module^40^.

**Figure 1:**
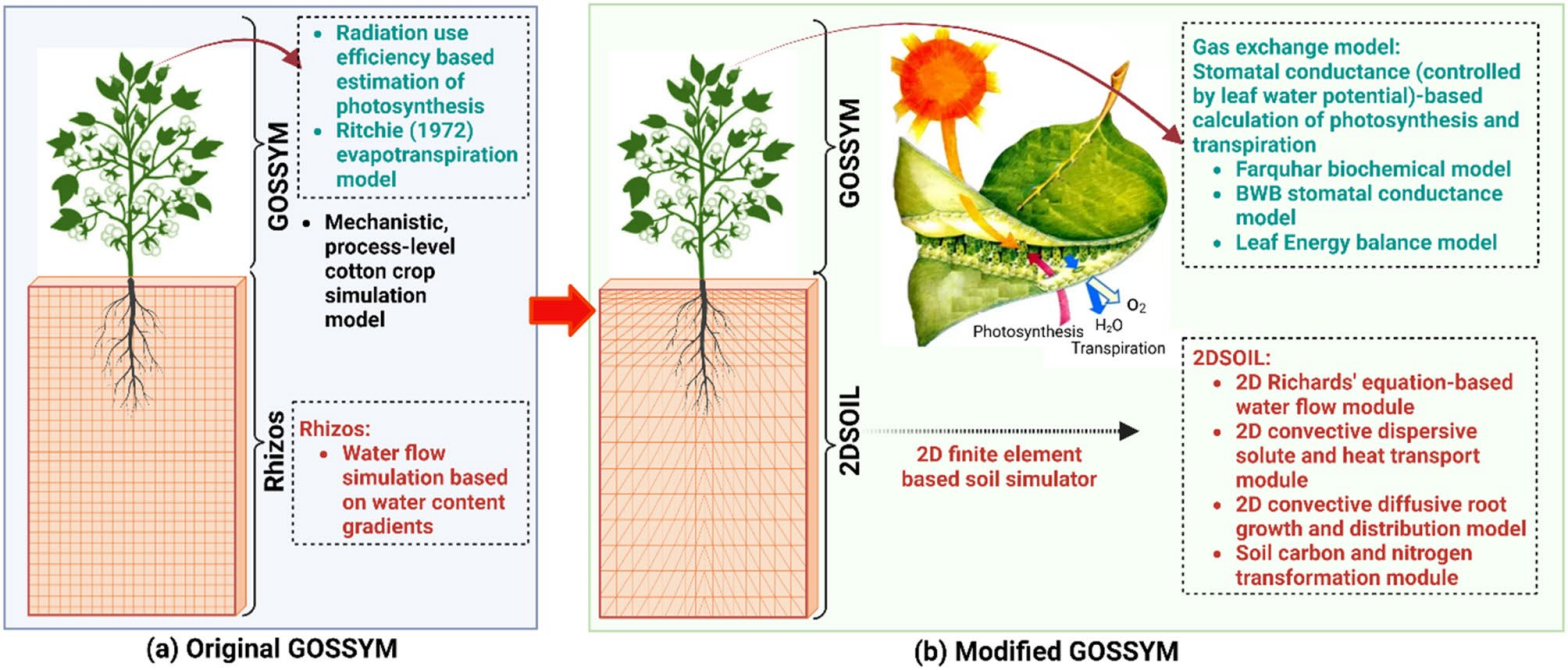
Schematic representation of improvements made to the original GOSSYM. (**a**) Original GOSSYM refers to the GOSSYM model with radiation use the efficiency-based estimation of photosynthesis, Ritchie's evapotranspiration model, and Rhizos (two-dimensional (2D) gridded soil model that simulates below-ground processes daily). (**b**) Modified GOSSYM refers to the original GOSSYM after improvements for the photosynthesis, transpiration, and below-ground process modeling components. Modeling of photosynthesis and transpiration are improved by incorporating the Farquhar biochemical model, Ball, Woodrow, and Berry (BWB) stomatal conductance model, and leaf-level energy balance model (gas exchange model). The below-ground process modeling is improved by incorporating 2DSOIL (2D finite element-based soil simulator). The primary modeling components in 2DSOIL are presented in the lower right box (**b**).

This report discusses the improvements made to photosynthesis, transpiration, and soil process simulations in GOSSYM and presents the developed model's capabilities based on illustrative examples with measured data. Photosynthesis and transpiration are improved by incorporating a stomatal conductance-based calculation of photosynthesis and transpiration (using the biochemical model for photosynthesis by Farquhar^26^, stomatal conductance model developed by Ball, Woodrow, and Berry (BWB)^36^, and the leaf-level energy balance model^37,38^ proposed by Kim and Lieth^27^ referred to as the 'gas exchange model' hereafter). Details of the gas exchange model can be found in the Supplementary information. The GOSSYM's prediction of below-ground processes is improved by incorporating the 2DSOIL model. All the processes simulated using Rhizos in GOSSYM are replaced with 2DSOIL (Fig. 1). The version of GOSSYM without any modification is referred to as 'original GOSSYM,' and GOSSYM after its integration with the 2DSOIL and gas exchange model is referred to as 'modified GOSSYM' hereafter (Fig. 1).

### Integration of gas exchange model and 2DSOIL model into GOSSYM.

**Figure. 2:**
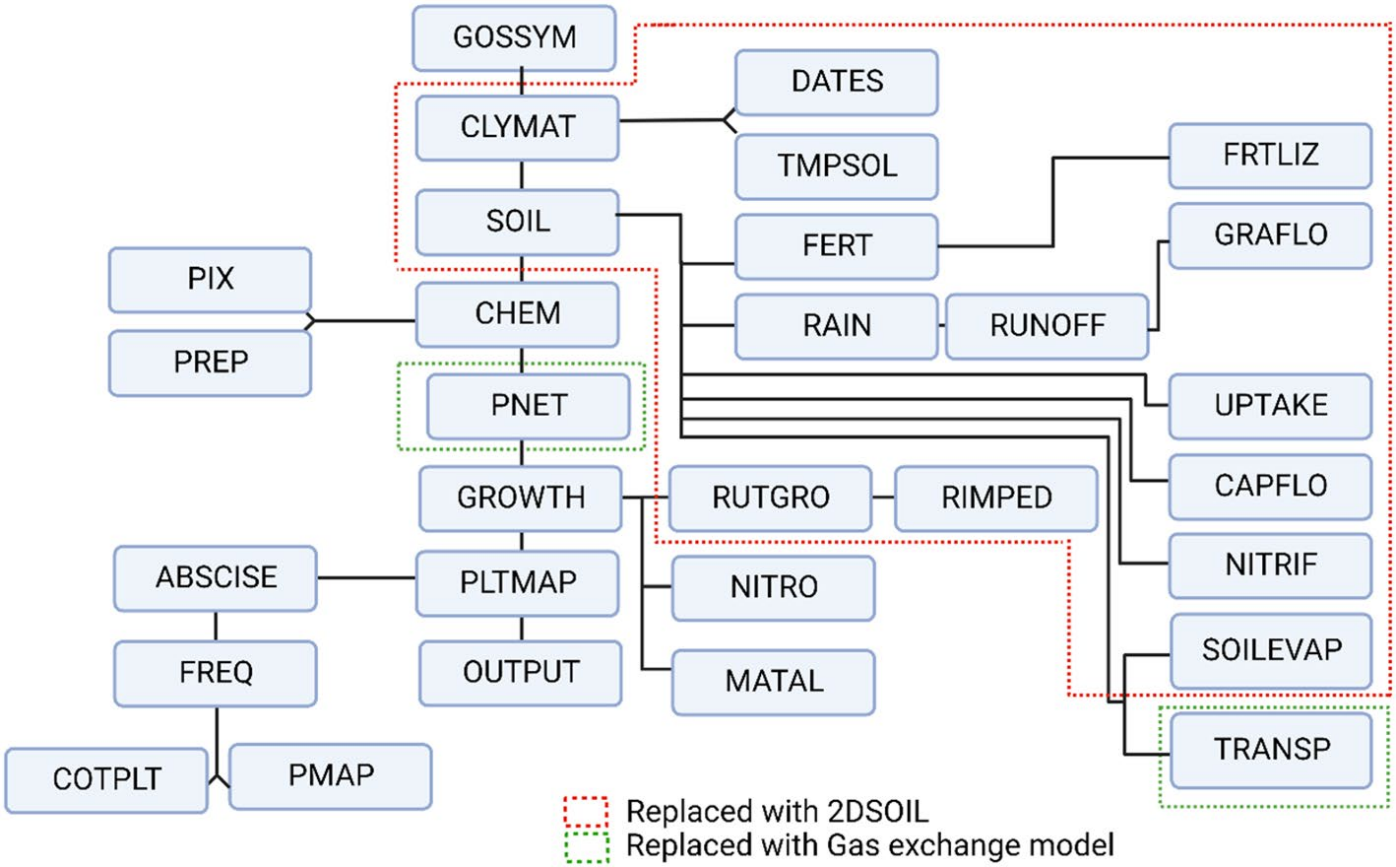
The general organization of different processes in GOSSYM. The process subroutines in GOSSYM replaced with 2DSOIL and the gas exchange model are marked in red and green boxes, respectively.

The organization of the original GOSSYM model and different simulated processes are shown in Fig. 2. GOSSYM is the main program that calls all the process subroutines^46^. CLYMAT reads the weather information, and TMPSOL calculates the soil temperature. The SOIL routine (Rhizos) includes all soil processes such as fertilization (FERT, FRTLIZ), rainfall (RAIN), runoff (RUNOFF), gravity flow (GRAFLO), root water uptake (UPTAKE), mineralization of organic matter, urea and their conversions (NITRIF). It also includes root growth (RUTGRO), the effect of bulk density on root elongation (RIMPED), and evapotranspiration calculation (SOILEVAP, TRANSP). The other subroutines simulate the impact of chemical growth regulators (CHEM) such as PIX and PREP, daily dry matter production (PNET), N stress calculation (NITRO), dry matter distribution to plant organs (GROWTH), morphogenetic processes, and the abortion of leaves and fruit (PLTMAP)^6^. GOSSYM is improved by incorporating the gas exchange model (by replacing the subroutines marked in the green box in Fig. 2). In addition to this, the soil and related processes in GOSSYM (Rhizos) (denoted in the red box in Fig. 2) are improved by incorporating the 2DSOIL model.

The GOSSYM model runs on a daily time step scale, and 2DSOIL runs on a sub-daily time step, with each process having separate time step requirements. The modular structure of 2DSOIL facilitates integrating new modules. The integration of GOSSYM and the gas exchange model with 2DSOIL requires the passing of state variables. The state variables passed from the GOSSYM to 2DSOIL are (a) carbon allocated to the roots, (b) LAI, (c) potential transpiration, and (d) plant N demand. Major state variables passed from 2DSOIL to GOSSYM are (a) leaf water potential, (b) average soil water potential in the root zone, (c) N uptake by the roots, (d) potential root growth, (e) carbon used for the root growth, and (f) root weight. Since 2DSOIL has sub-daily time steps, the variables passed from 2DSOIL to GOSSYM are averaged or cumulated over one day. The variables passed from 2DSOIL and GOSSYM to the gas exchange model are (a) photosynthetically active radiation, (b) air temperature, (c) CO_2_ concentration, (d) vapor pressure deficit, (e) wind speed, (f) LAI, (g) leaf water potential and (h) plant N status. GOSSYM and 2DSOIL are written in Fortran programming language, and the gas exchange model is written in C ++ . The program can be compiled and run using any standard FORTRAN compiler.

### Model evaluation and discussion

The improved capabilities of the modified GOSSYM are illustrated through two testing examples. Example 1 is based on a field experiment, and example 2 is based on the experimental study conducted in sunlit Soil–Plant–Atmospheric Research (SPAR) chambers at Starkville, Mississippi. In example 2, the experiment was carried out for two CO_2_ concentration levels (360 ppm and 720 ppm) and three temperature controls. One temperature control mimicked the outdoor temperature (temperature: 1995). The other two conditions were controlled to outdoor temperature plus two degrees (temperature: 1995 + 2 °C) and minus two degrees (temperature: 1995 – 2 °C). The details of the experiments and the scenarios considered for modeling in example 2 are given in Table 1. The model setup, data used for both examples and performance indices used for model evaluation are discussed in the Method section. Simulation results from the original and modified GOSSYM are compared for both examples.

**Table 1:** Details of the experiment and modeling scenarios considered in illustrative example 2.

Scenario	Description
CO_2_: 360 ppm, temperature: 1995	Temperature used in this scenario mimicked the outdoor temperature observed in the year 1995
CO_2_: 720 ppm, temperature: 1995	
CO_2_: 360 ppm, temperature: 1995 + 2 °C	Temperature used in this scenario is the temperature observed in the year 1995 plus 2 °C
CO_2_: 720 ppm, temperature: 1995 + 2 °C	
CO_2_: 360 ppm, temperature: 1995 – 2 °C	Temperature used in this scenario is the temperature observed in the year 1995 minus 2 °C
CO_2_: 720 ppm, temperature: 1995 – 2 °C	

**Figure 3:**
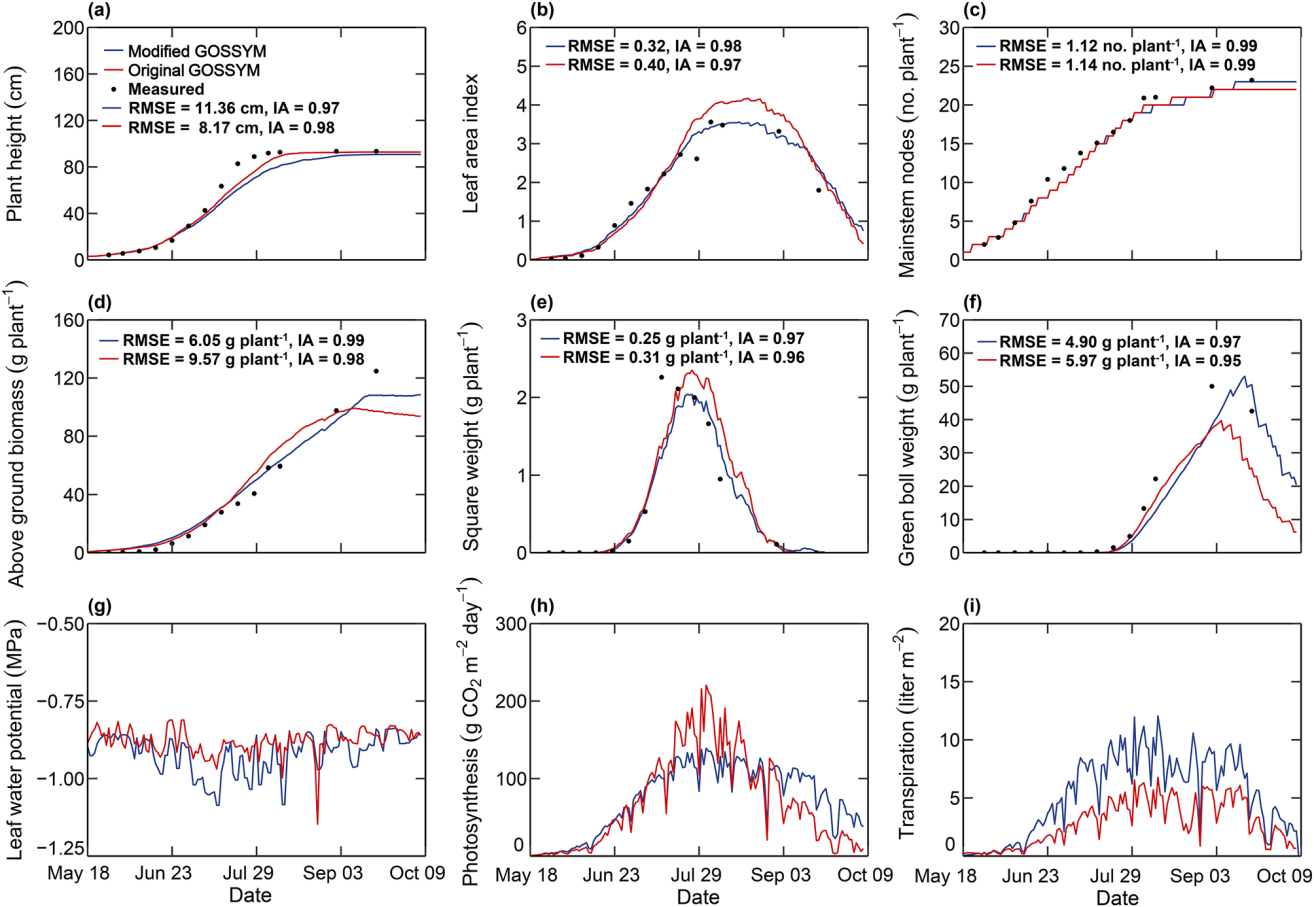
Measured and simulated (using modified GOSSYM and original GOSSYM) (**a**) plant height, (**b**) leaf area index, (**c**) the number of mainstem nodes, (**d**) total above-ground biomass, (**e**) weight of squares, (**f**) green boll weight, and simulated (**g**) leaf water potential, (**h**) net photosynthesis, and (**i**) transpiration in example 1. The legend for this figure is presented in the top-left corner of panel (**a**) and applies to all panels in the figure.

Figures 3a–f illustrate the measured and simulated plant height, LAI, number of mainstem nodes, total plant biomass, the weight of squares, and green boll in illustrative example 1**.** Except for plant height, all other measured plant growth components (LAI, number of mainstem nodes, total biomass, and square and green boll weight) were better simulated using modified GOSSYM as shown by the lower root mean square error (RMSE) and higher index of agreement (IA) (Fig. 3). Simulated leaf water potential, net canopy photosynthesis, and transpiration are shown in Fig. 3g–i. The simulated cumulative net photosynthesis was 130.6 and 145.1 g C plant^−1^ for the original and modified GOSSYM, respectively. The simulated cumulative transpiration was 413.5 and 796.4 mm plant^−1^ for the original and modified GOSSYM, respectively. In example 1, the difference in the overall plant growth and development (in terms of plant height, LAI, biomass, cotton fiber yield, etc.) using the original and modified GOSSYM was mainly due to the difference in simulated leaf water potential and average soil water potentials, photosynthesis, and transpiration as a result of the integration of 2DSOIL and the Farquhar gas exchange model.

Leaf water potential simulation in GOSSYM is a function of average soil water content in the root zone^47^. The difference in average soil water content in the root zone simulated using the original GOSSYM (using Rhizos, with an average value of − 0.09 MPa) and modified GOSSYM (using 2DSOIL, the average value of − 0.19 MPa) resulted in the differences in simulated leaf water potential variation in example 1. The leaf water potential simulated using the original GOSSYM was higher (average of − 0.87 MPa) than the modified GOSSYM (average of − 0.92 MPa) (Fig. 3g). Leaf and stem growth water stress in GOSSYM is simulated as a function of leaf water potential^47,48^. This decrease in leaf water potential increased leaf growth water stress and resulted in lower leaf area and LAI using modified GOSSYM compared to the original GOSSYM (Fig. 3b). The decrease in the leaf area resulted in a reduction of daily net photosynthesis simulated using modified GOSSYM from July 15th to August 21st, 1992 (Fig. 3h). Towards the end of August, the increase in the leaf water potential reduced the water stress for leaf and shoot growth which increased the daily net photosynthesis. An increase in the leaf water potential and the net photosynthesis towards the end of August resulted in more carbon available for growth (Fig. 3h). This reduced the time interval for the formation of nodes and caused an increase in number of nodes using the modified GOSSYM (Fig. 3c). Total plant height is simulated as a function of a total number of pre-fruiting nodes and nodes on first vegetative branch (which forms main stem in cotton)^18^. The total number of mainstem nodes for the original and modified GOSSYM was 21 and 23, respectively. The increase in the mainstem nodes towards the end of August resulted in an increase in plant height in case of modified GOSSYM (99 versus 103 cm in original versus modified GOSSYM, respectively). An increase in net photosynthesis towards the later period resulted in a higher cotton fiber yield prediction from modified GOSSYM (1156 kg ha^−1^) compared to the original GOSSYM (1057 kg ha^−1^).

Transpiration was higher for modified GOSSYM as compared to the original GOSSYM (Fig. 3i). This is due to the differences in the method of transpiration estimation and the soil water flow simulations in both models. Previous studies using original GOSSYM also reported an underestimation of evapotranspiration using Ritchie's^4^ model^17^. In the modified GOSSYM, once the potential transpiration is determined, the simulated transpiration is calculated based on the root growth and water uptake model by Acock and Trent^49^. The transpiration is a function of the difference in soil and leaf water potential and is reduced based on soil-to-root, root, and xylem hydraulic resistance^50^. In the original GOSSYM, the water uptake is estimated based on root density in the cells, age distribution of roots, and water status of cells as characterized by soil water diffusivity^30^. Simulated days of the first square, first bloom, and first open boll using the original GOSSYM and modified GOSSYM were found to be the same. Both models accurately predicted the first day of the square. The simulated date of the first bloom and first open boll was the same for both models but was predicted to be one day before the measured date. Total cotton fiber yield at harvest was also better simulated by modified GOSSYM with an absolute percentage error of 1.3% and 7.3% for modified and original GOSSYM, respectively.

**Figure 4:**
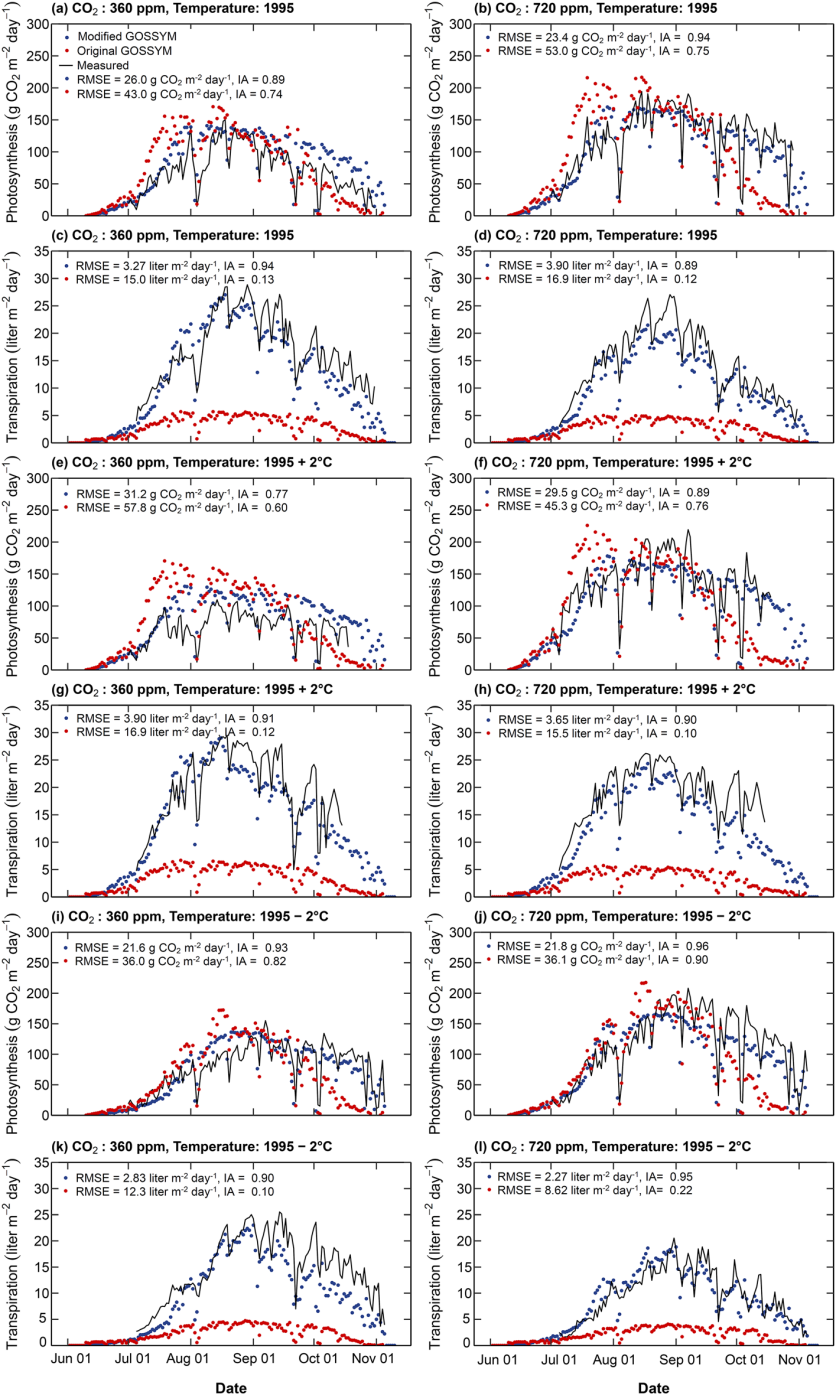
Measured and simulated (using modified and original GOSSYM) net daily photosynthesis (**a**, **b**, **e**, **f**, **i**, and **j**) and transpiration (**c**, **d**, **g**, **h**, **k**, and **l**) for the scenario: CO_2_: 360 ppm, temperature: 1995 °C (**a, c**), CO_2_: 720 ppm, temperature: 1995 °C (**b**, **d**), CO_2_: 360 ppm, temperature: 1995 + 2 °C (**e**, **g**), and CO_2_: 720 ppm, temperature: 1995 + 2 °C (**f**, **h**) and CO_2_: 360 ppm, temperature: 1995 + 2 °C (**i**, **k**), and CO_2_: 720 ppm, temperature: 1995 + 2 °C (**j**, **l**) (Example 2). Temperature: 1995 refers to temperature control (in the soil plant atmospheric research (SPAR) chambers) that mimicked the outdoor temperature. Temperature: 1995 + 2 °C and temperature: 1995 – 2 °C refer to temperatures controlled to outdoor temperature plus two degrees (temperature: 1995 + 2 °C) and minus two degrees (temperature: 1995 – 2 °C), respectively. IA is the index of agreement, and RMSE is the root mean square error. The legend for this figure is presented in the top-left corner of panel (**a**) and applies to all panels in the figure.

Measured and simulated daily photosynthesis and transpiration for all the scenarios in example 2 are presented in Fig. 4**.** For all the temperature treatments, the measured net photosynthesis was higher for elevated CO_2_ (720 ppm) than ambient CO_2_ (360 ppm) conditions (an increase of 46.5%, 60.23%, and 99.1% for temperature: 1995 – 2 °C, 1995, and 1995 + 2 °C, and respectively). The increase in photosynthesis with an increase in CO_2_ concentration is mainly due to the rise in ribulose-1,5-bisphosphate (RuBP) carboxylase /oxygenase (Rubisco) activity^51,52^. A higher increase in net photosynthesis was observed in the scenario with temperature 1995 + 2 °C followed by 1995 and 1995 – 2 °C temperature scenarios. The increase in net photosynthesis is a function of temperature during critical growth stages in cotton crops, significantly influencing crop development. Average temperatures of 28 °C and 32 °C are considered the optimum and maximum cardinal temperatures for cotton growth^53^. In this example, the number of days with optimum temperature conditions was 47, 16, and 0 for the temperature scenario; 1995 + 2 °C, 1995, and 1995 – 2 °C, respectively.

**Figure 5:**
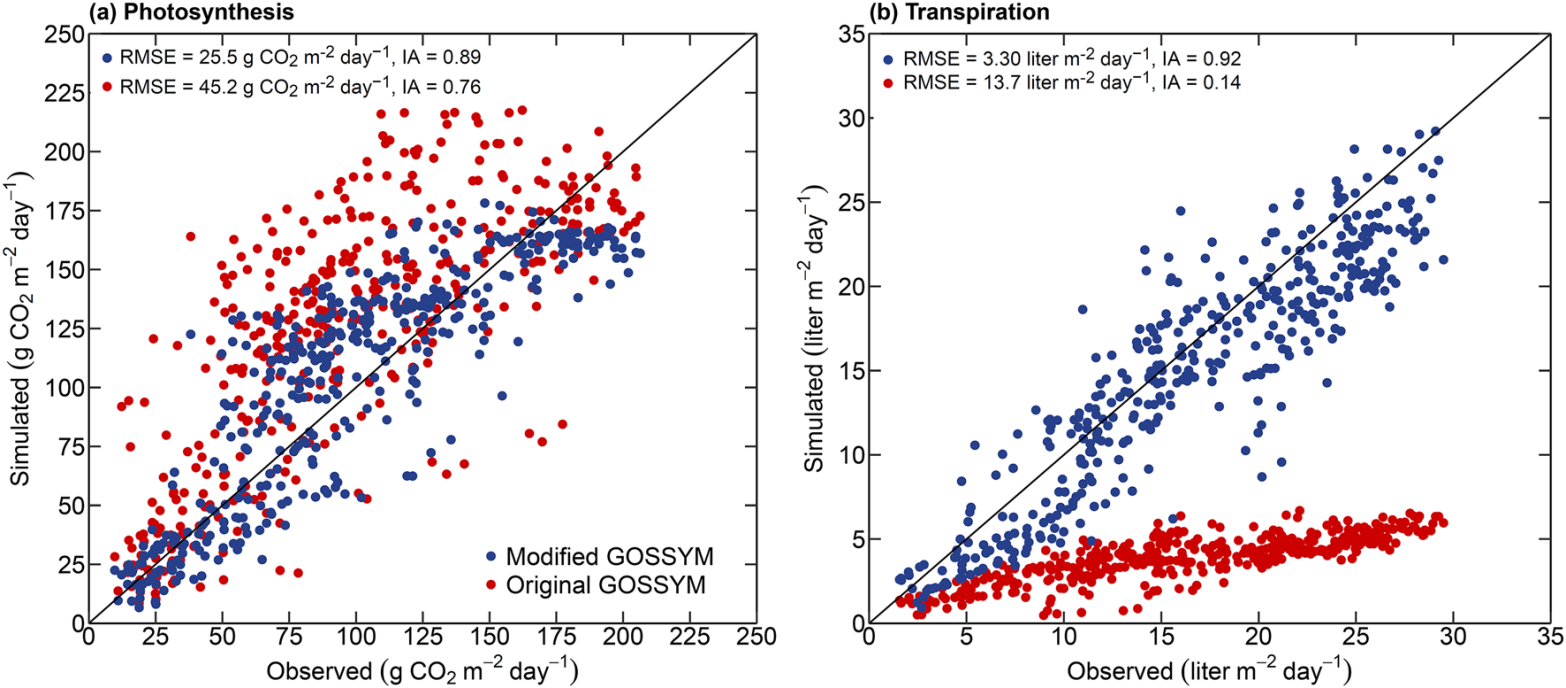
Measured and simulated net daily photosynthesis (**a**) and transpiration (**b**) for all the six scenarios (CO_2_: 360 ppm, temperature: 1995, CO_2_: 720 ppm, temperature: 1995, CO_2_: 360 ppm, temperature: 1995 + 2 °C, CO_2_: 720 ppm, temperature: 1995 + 2 °C, CO_2_: 360 ppm, temperature: 1995 – 2 °C, CO_2_: 720 ppm, temperature: 1995 – 2 °C in example 2 (Table 1). RMSE is the root mean square error, and IA is the index of agreement.

The root mean square error (RMSE) and index of agreement (IA) between simulated and measured net photosynthesis and transpiration using the original and modified GOSSYM are presented in Fig. 4**.** Measured and simulated photosynthesis and transpiration for all six scenarios along with RMSE and IA are combinedly shown in Fig. 5. For all the simulated scenarios, a higher IA and a lower RMSE between simulated and measured daily photosynthesis was observed using modified (average IA = 0.89 and RMSE = 25.5 g CO_2_ m^−2^ day^−1^) compared to the original GOSSYM (average IA = 0.76 and RMSE = 45.2 g CO_2_ m^−2^ day^−1^). In all scenarios, the original GOSSYM overestimated net photosynthesis in the initial crop growth period, followed by underestimating in the later crop period. A similar observation was made in example 1. In the original GOSSYM, a correction factor (pnetcor) is incorporated into the gross photosynthesis equation to account for atmospheric CO_2_ concentration. The model responds to higher atmospheric CO_2_ conditions by increasing photosynthesis with reference to a standard CO_2_ value of 320 ppm^1,54,55^. In example 2, the pnetcor value was thus 1.3. In the modified GOSSYM, the effect of atmospheric CO_2_ levels is mechanistically accounted for in the photosynthesis estimation as a function of intercellular CO_2_ partial pressure and stomatal conductance, which are linked to CO_2_ partial pressure at the leaf surface.

For all the CO_2_ and temperature scenarios, transpiration was better simulated using modified GOSSYM (average IA = 0.92 and RMSE = 3.30 L m^−2^ day^−1^) compared to original GOSSYM (average IA = 0.14 and RMSE = 13.73 L m^−2^ day^−1^) (Fig. 5b). Measured and simulated transpiration in the elevated CO_2_ condition was lower than the ambient CO_2_ condition for all temperature treatments. A decrease in water uptake with an increase in the CO_2_ concentration in most plant species is associated with elevated CO_2_-induced stomatal closure^56–58^. Original GOSSYM was found to be highly underestimating transpiration. In the original GOSSYM, transpiration is calculated using Eq. (1).

1 $$E_{T} = \left[ {\frac{\Delta }{\gamma } R_{no} + 0.262*\left( {1 + 0.0061u} \right)\left( {e_{o} - e_{a} } \right) } \right]\left( {\frac{\Delta }{\gamma } + 1} \right)^{ - 1}$$

where, $E_{T}$ is the potential transpiration, $\Delta$ is the slope of the saturation vapor pressure curve at mean temperature, $\gamma$ is the constant of the wet and dry bulb psychrometric equation, $R_{no}$ is the net solar radiation above the canopy, $u$ is the wind speed, $e_{o}$ is the saturation vapor pressure at mean air temperature and $e_{a}$ is the mean vapor pressure at minimum daily temperature^4^. The model uses minimum air temperature as dew point temperature, which is observed to function well in humid conditions but not semi-arid conditions^17^. In the modified GOSSYM, relative humidity is used to estimate the vapor pressure deficit. In contrast, in the original GOSSYM, relative humidity is not read as an input, and the vapor pressure deficit is calculated as a function of maximum and minimum temperature. In example 2, relative humidity ranged from 40 to 60%. The original GOSSYM was not able to reflect the impact of the relative humidity on the transpiration estimated, which has resulted in relatively lower predictions for transpiration. Clouse^59^ and Staggenborg et al.^17^ also observed an underprediction of transpiration by 18% and 26% using original GOSSYM^13^. It was also observed that the transpiration estimated using the original GOSSYM responded less to CO_2_ concentration. This is due to the determination of potential evapotranspiration in original GOSSYM using the model developed by Ritchi^4^ (Eq. 1) that does not consider the impact of CO_2_ levels.

**Figure 6:**
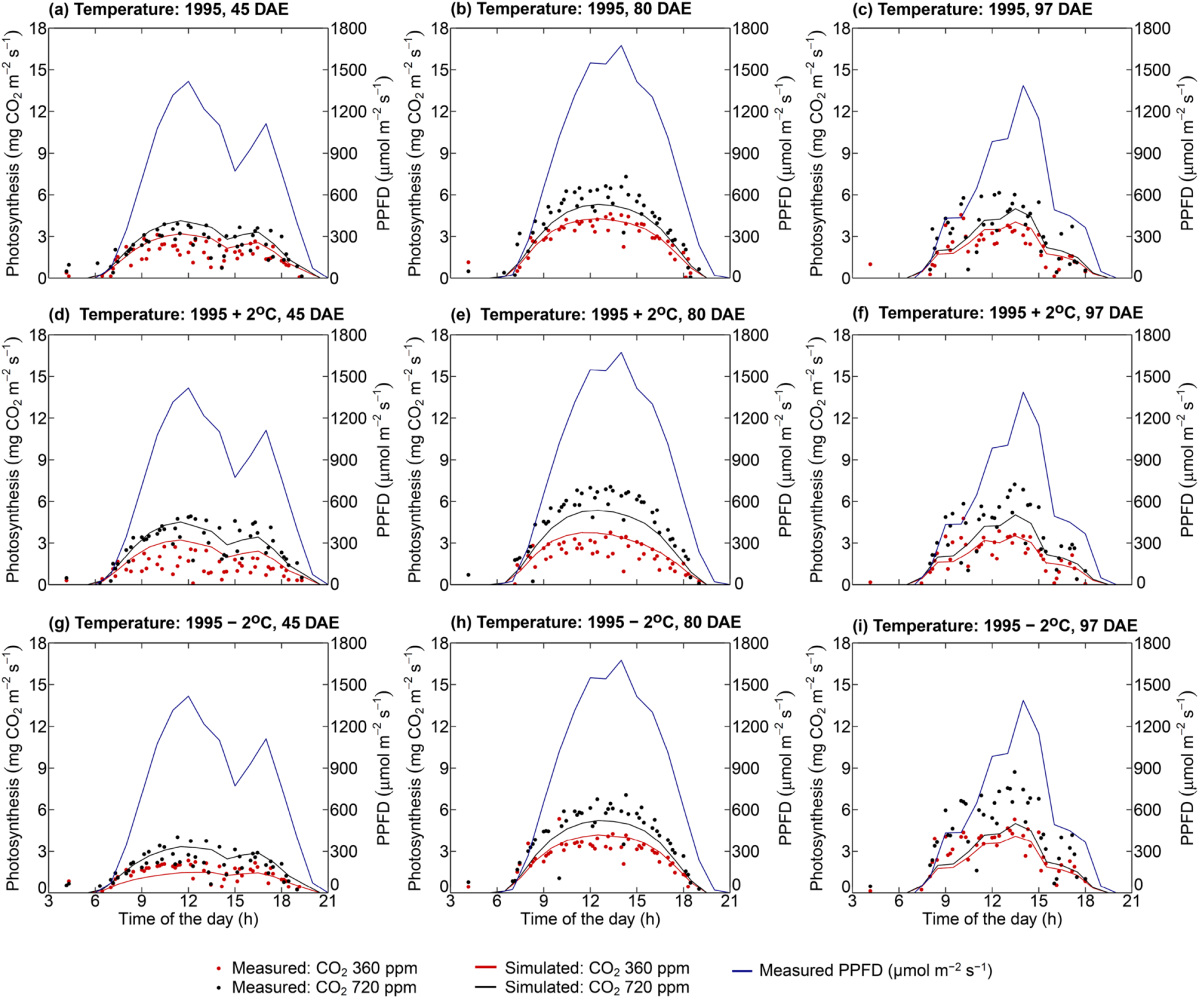
Photosynthetic photon flux density (PPFD), measured and simulated photosynthesis on 45 (**a**, **d**, **g**), 80 (**b**, **e**, **h**), and 97 (**c**, **f**, **i**) days after emergence (DAE) using modified GOSSYM for two CO_2_ levels (360 ppm and 720 ppm) for three temperature controls (Temperature: 1995, Temperature: 1995 + 2 °C, temperature: 1995 – 2 °C). Temperature: 1995 refers to temperature control (in the soil plant atmospheric (SPAR) chambers) that mimicked the outdoor temperature. Temperature: 1995 + 2 °C and temperature: 1995 – 2 °C refer to temperatures controlled to outdoor temperature plus two degrees (temperature: 1995 + 2 °C) and minus two degrees (temperature: 1995 – 2 °C), respectively.

Radiation use efficiency-based growth estimation in the original GOSSYM was incapable of simulating sub-daily levels of photosynthesis. But all the soil processes, photosynthesis, and transpiration can be simulated at hourly or sub-daily time steps in the modified GOSSYM. Modified GOSSYM's capability in simulating diurnal photosynthesis is illustrated by considering three representative dates after emergence (DAE) (two partly cloudy days (45 and 97 DAE) and a sunny day (80 DAE)). The average daily light integral for the partially clouded dates and sunny days was 19.57 MJ m^−2^ day^−1^ and 23.94 MJ m^−2^ day^−1^. The measured and simulated diurnal photosynthesis and photosynthetic photon flux density (PPFD) for the three representative days for all the scenarios presented in Table 1 are shown in Fig. 6. Simulation results show that modified GOSSYM well predicted (with average IA = 0.94, RMSE = 0.79 mg CO_2_ m^−2^ s^−1^) the daily fluctuation in the photosynthesis. This shows the modified GOSSYM's capability of simulating the sub-daily levels of photosynthesis.

In addition to the improvement of the original GOSSYM due to the incorporation of 2DSOIL and an energy balance and biochemical-based simulation of photosynthesis and transpiration, the changes in model features associated with these components also improved the simulation capabilities of the modified GOSSYM. The two models' spatial and temporal discretization, model settings, and governing equations used to simulate soil, plant, and atmospheric processes are different. The original GOSSYM runs on a daily time step, whereas in the modified GOSSYM, the soil processes (using 2DSOIL) and gas exchange routine run on sub-daily time steps. In 2DSOIL, the time step discretization depends on several criteria, including the numerical solution and implementation of boundary conditions. The van Genuchten model^60^ describes the relationship between the soil hydraulic properties in the modified GOSSYM. In contrast, the original GOSSYM uses the Marani soil–water release equation for water movement.

## Summary and conclusion

A coupled biochemical energy balance model for photosynthesis and stomatal conductance and a two-dimensional finite element soil model (2DSOIL) is integrated into the cotton model, GOSSYM, to improve its predictions of photosynthesis, transpiration, and below-ground processes. The performance of the modified model is evaluated based on two illustrative examples, and it is observed that the new model resulted in an improved simulation of crop growth and development processes. The new model can also simulate photosynthesis, transpiration, and soil processes at sub-daily time steps, which was not a feature of the previous version of GOSSYM. The developed model can be used for cotton crop simulations under varying soil water, temperature, CO_2_ dynamics, and nutrient stresses. To illustrate the model's potential in simulating the integrated effect of varying CO_2_, temperature, and soil water and nutrient conditions, a more detailed analysis can be carried out based on controlled CO_2_, water, nutrient, and temperature settings experiments.

The latest IPCC report (IPCC, 2021) shows that atmospheric CO_2_ is increasing and has reached an annual average value of 410 ppm. The air temperature was also 1.09º C higher in 2011–2020 than in 1850–1900. In the future, several climatic features are predicted to continue to alter from their historical norms. Our understanding of the impacts of climate change on crop growth and development is based on controlled small-scale experimental studies. Improving existing crop simulation models based on these understandings is crucial. The integration of 2DSOIL and the gas exchange model at the process level is an update to the GOSSYM model. The modified GOSSYM facilitates simulating varying possible climate scenarios and can assist policymakers in developing adaptation strategies.

## Methods

### Data used in the illustrative example 1 and the model setup

Example 1 is based on a field experiment with the cotton cultivar DPL-90. The experiment was carried out in Stoneville, Mississippi (33° 15′ 9.36″ N, 90° 32′ 43.44″ W) in 1992. The cotton crop was cultivated with a row spacing of 96.5 cm and a plant density of 10.19 plants m^−2^. Cotton was grown in rainfed conditions (no irrigation applied). Crop emergence and harvest dates were May 18th, 1992, and October 7th, 1992. Fertilizer was applied on April 6th, 1992 (28.02 kg N ha^−1^ of ammonium and 28.02 kg N ha^−1^ of nitrate) and June 6th, 1992 (22.4 kg N ha^−1^ of ammonium and 22.4 kg N ha^−1^ of nitrate). The number of mainstem nodes, plant height, LAI, above-ground biomass, weight of squares, and green bolls were measured. The dates of the first square, first bloom, and first open boll and total cotton fiber yield after harvesting were recorded.

For simulating example 1, a soil profile of 2 m below the plant and a width equal to row spacing (96.52 cm) was considered. The soil profile was divided into three soil layers (0–19 cm, 19–42 cm, and 42–200 cm) with varying soil properties. In GOSSYM, 50 calibration parameters modify the simulation of the growth and development of different cotton varieties. Among these, parameters for developmental time (time to the first square, first square to bloom, bloom to open boll), plant height, and node initiation on the main stem and vegetative branches are the major crop parameters used for model calibration^15,61^. Calibrated crop model parameters for the DPL-90 variety were obtained from previous modeling studies for the same cotton variety using original GOSSYM^15,61,62^. Only the parameters controlling the time to the first square and time to first bloom (developmental time) were manually calibrated to minimize the difference between the measured and simulated dates of the major crop events (date of the first square, first bloom). The parameter controlling time to the first square was changed from 0.95 to 0.90. This decreased the date of the first square by one day. The parameter controlling the time to first flower was changed from 1.0 to 1.05, increasing the time to first bloom by three days. Since there were no differences in the modeling of developmental time in both models, the same set of calibration parameters was used in the original and modified GOSSYM. The cotton parameters used for the gas exchange module were obtained from Baker et al.^63^ Kim and Lieth^27^, and Medlyn et al.^64^. These parameters are given in Supplementary Table S1. The atmospheric CO_2_ concentration used in the simulation was 350 ppm which was present during the experiment.

For the original and modified GOSSYM, the atmospheric boundary condition was considered at the surface with prescribed daily solar radiation, minimum temperature, maximum temperature, rainfall, and wind speed. A free drainage boundary was used at the bottom of the soil profile. In the original GOSSYM, the total soil domain (200 m deep and 96.52 wide) was divided into 40 rows and 20 columns. The dimension of each of the cells considered was 5 cm deep, 4.8 cm wide, and 1 cm thick. In modified GOSSYM, the domain was divided into finite elements. The total nodes, elements, and boundary points used in this study were 330, 290, and 22, respectively. The finite element domain had a varying element size and nodal spacing with relatively small nodal spacing at locations with more significant hydraulic gradients (closer to the soil surface and location of the plant). The spatially varying information about the residual N and C concentration in the soil, soil temperature, and initial pressure heads were entered as nodal information in the model. For the original GOSSYM, soil hydraulic properties used were diffusivity at -15,000 cm potential, volumetric water content at − 15,000 cm potential, hydraulic conductance, saturated water content, water content at field capacity, residual water content, water content at air-dry condition, bulk density, percentage of sand and clay. For the modified GOSSYM, the soil hydraulic properties used were the residual water content, saturated water content, hydraulic conductivity, and van Genuchten parameters (alpha and η)^60^.

### Data used in the illustrative example 2 and the model setup

Example 2 is based on the experimental study conducted in sunlit Soil Plant Atmospheric Research (SPAR) chambers at Starkville, Mississippi (33° 28′ 12″ N, 88° 46′ 54″ W) in 1995^65^. The experiment was carried out for two CO_2_ concentration levels (360 ppm and 720 ppm) and three temperature controls. One temperature control mimicked the outdoor temperature (temperature: 1995). The other two conditions were controlled to outdoor temperature plus two degrees (temperature: 1995 + 2 °C) and minus two degrees (temperature: 1995 – 2 °C). Cotton was planted on June 6th, 1995. Cotton plants (DPL 51) in all six chambers, each 2.5 m tall, 2 m long, and 1.5 m wide, but with 0.5 m growing area were arranged in three rows with row spacing of 40 cm. Plant density was 15 plants m^−2^. Fine sand was filled in the chamber up to a depth of 90 cm. The cotton plants were well watered, and enough nutrients were applied to avoid water or nutrient-stressed conditions. Canopy-level photosynthesis was estimated using a mass balance approach, which was facilitated by the airtight nature of the plexiglass chamber containing the plants, ducts, and cooling system in the SPAR setup^66^. To estimate net CO_2_ exchange rates, the mass of CO_2_ injected into the chamber to maintain treatment setpoints (360 ppm and 720 ppm) and the mass of CO_2_ lost via leakage were quantified. The mass of CO_2_ injected was estimated based on the duration of the valve openings through which CO_2_ was injected, while leakage was estimated by conducting a leakage test every night^66,67^. Transpiration was measured based on the amount of water condensed by the air conditioner, providing a direct measure of transpiration^66,68,69^.

For example 2, the input files required for simulating the SPAR experiments using the original and modified GOSSYM were created similarly to example 1. A soil profile of 0.9 m below the plant and a width equal to row spacing (40 cm) was considered. Calibrated crop model parameters for the DPL-51 variety were obtained from previous modeling studies for the same cotton variety^15^. Only the parameters controlling the pre-fruiting branch node time interval were manually calibrated for this study. Increasing this parameter from 1.25 to 1.5 increased the time for initiating a pre-fruiting node and increased the time to the first square by two days. Gas exchange model parameters given in Supplementary Table S1 were used. The measured and simulated photosynthesis and transpiration (using the original and modified GOSSYM) were compared and analyzed.

Experiments discussed in examples 1 and 2 were performed in accordance with the relevant guidelines and regulations.

### Evaluation of the model performance

The performance of the modified and original GOSSYM is evaluated based on the root mean square error (RMSE) and Willmott's index of agreement (IA)^70^. Simulated and measured refers to simulated and measured data at a point in time during the growing season, and n is the number of observed data. Lower RMSE values indicate the closeness of the measured values with the simulated ones. IA reflects the degree to which the simulated variate accurately estimates the measured variate. A value of 1.0 indicates a perfect agreement, and 0.0 means no agreement^70^.

## Supplementary Information

Supplementary Information.

## Data Availability

The data supporting the findings of this study are available within the article and the supplementary materials.

## Code availability

The latest version of the GOSSYM source code after its integration with the 2DSOIL and gas exchange model (developed in the current study) can be found in USDA-ARS-ACSL/GOSSYM-2DSOIL: The Gossym cotton model that has been merged with 2DSOIL (https://github.com/USDA-ARS-ACSL/GOSSYM-2DSOIL). The source code of the GOSSYM model before its integration with 2DSOIL and gas exchange model is available at https://github.com/USDA-ARS-ACSL/GOSSYM_Orig. There are no restrictions for accessing the source code.
