# Supplementary material for: Improving the cotton simulation model, GOSSYM, for soil, photosynthesis, and transpiration processes
Source: Sci Rep. 2023 May 5;13:7314. doi: 10.1038/s41598-023-34378-3 (PMC10163017; doi:10.1038/s41598-023-34378-3)
Supplement: Supplementary file 1 — Supplementary Information. [file 41598_2023_34378_MOESM1_ESM.docx]

**Supplementary Information**

## Gas exchange model

Three sets of equations/models are used to describe the coupled photosynthesis, stomatal conductance, and transpiration model: (a) biochemical model for photosynthesis^1^, (b) the calculation of stomatal conductance to water vapor based on stomatal conductance model (BWB model)^2^, and (c) energy balance model.

### **The biochemical model for photosynthesis by Farquar, Von Caemmerer, and Berry (FvCB)**^1^

The biochemical model for photosynthesis by Farquar, Von Caemmerer, and Berry^1^ is obtained using **equation (S1).**

$A=v_{C}-0\cdot5v_{0}-R_{d}=\min\left\{ A_{C},A_{j},A_{P} \right\}-R_{d}$ (S1)

$A$ is the net photosynthesis rate ($\mu mol m^{-2} s^{-1})$, $v_{c}$ is the carboxylation rate $(\mu molm^{-2} s^{-1})$, $v_{o}$ is oxygenation rate ($\mu mol m^{-2} s^{-1})$, $R_{d}$ is mitochondrial respiration in the light $(\mu molm^{-2} s^{-1})$, $A_{c}$ is the rubisco- limited CO_2_ assimilation rate $(\mu molm^{-2} s^{-1})$, $A_{j}$ is the electron transport limited CO_2_ assimilation rate $(\mu molm^{-2} s^{-1})$, $A_{p}$ is the triose phosphate utilization- limited CO_2_ assimilation rate $(\mu molm^{-2} s^{-1})$.

$A_{c}=V_{cmax}\frac{Ci-\Gamma*}{C_{i}+K_{c}(1+\frac{O}{k_{0}})}$ (S2)

$A_{c}$ is determined using **equation (S2),** where $V_{cmax,}$ is the maximum carboxylation rate $(\mu molm^{-2} s^{-1})$, $C_{i}$ is the intercellular CO_2_ partial pressure $(\mu bar)$, $\Gamma*$ is the CO_2_ compensation point in the absence of $R_{d}$ $(\mu bar)$, $K_{c}$ is the Michaelis-Menten constant of rubisco for CO_2_ (59.4 $\mu molm^{-2} s^{-1})$, O is the oxygen partial pressure (20500 Pa),$k_{0}$ is the Michaelis-Menten constant of rubisco for O_2_ (36$\mu molm^{-2} s^{-1})$.

$A_{j}= \frac{J(C_{i}-\Gamma*)}{4(C_{i}+2\Gamma*)}$ (S3)

$A_{j}$ is determined using **equation (S3)**, where $J$ is the electron transport rate $(\mu mol{electrons m}^{-2} s^{-1})$.

$Ap=3TPU$ (S4)

$A_{p}$ is determined using **equation (S4)**, where $TPU$ is the triose phosphate utilization rate $(\mu molm^{-2} s^{-1})$. The three processes occur concurrently in the chloroplast, but Farquhar et al.^1^ determined that the minimum of the three processes determines the net rate at any time.

### **Calculation of stomatal conductance to water vapor based on the stomatal conductance model (BWB model)**^2^

Stomatal conductance based on the BWB model is calculated using **equation (S5)**.

$g_{s}=b+mA\frac{h_{s}}{\left( \frac{C_{s}}{P_{a}} \right)}$ (S5)

Where $g_{s}$ is the stomatal conductance to water vapor $\left( molm^{-2} s^{-1} \right)$, $b$ is the minimum stomatal conductance to water vapor at the light compensation point $(0.0960 molm^{-2} s^{-1})$, $m$ is the empirical coefficient (15.0) for the sensitivity of $g_{s}$ to $A$, $C_{s}$, $h_{s}$. $C_{s}$ is CO_2_ partial pressure at the leaf surface $(\mu bar)$, and $h_{s}$is the relative humidity at leaf surface.

$g_{s}=b+mA\frac{h_{s}}{\left( \frac{C_{s}}{P_{a}} \right)}f(\Psi)$ (S6)

A modified version of **equation (S5) (equation (S6))** that incorporates the effect of leaf water status (f(Ψ)) on stomatal closure is used in GOSSYM ^3,4^.

### **Energy balance model**

The energy balance equation is used to estimate leaf temperature $(T_{L})$ as a function of stomatal conductance $\left( g_{s} \right),$boundary layer conductance $(g_{b})$ and other variables (air temperature, absorbed long and short-wave radiation, relative humidity).

$T_{L}=T_{a}+\frac{R_{abs}-\varepsilon\sigma T_{a}^{4}-\lambda g_{v}D/P_{a}}{c_{p}\left( g_{h}+g_{r} \right)+\lambda\left\{ \frac{\left( \frac{d}{dT}es\left( T_{a} \right) \right)}{P_{a}} \right\}g_{v}}$ (S7)

$T_{a}$ is the air temperature $(℃)$, $R_{abs}$ is the absorbed long wave and short wave radiation per surface leaf area $(Wm^{-2})$, $\varepsilon$ is leaf thermal emissivity (0.97), $\sigma$ is the Stefan-Boltzmann constant per surface area $(5.67 x {10}^{-8} Wm^{-2}K^{-4})$, $\lambda$ is the is the latent heat of vaporization at 25$℃$ $(44.0 kJ {mol}^{-1})$, $D$ is vapor pressure deficit of the ambient air $(kPa)$, $P_{a}$ is atmospheric pressure $(kPa)$, $c_{p}$ is the specific heat of the air $(29.3 J {mol}^{-1} C^{-1})$, $g_{h}$ is the heat conductance for the boundary layer per surface leaf area $(molm^{-2} s^{-1})$,$g_{r}$ is radiative conductance per surface leaf area $(molm^{-2} s^{-1})$, $es\left( T_{a} \right)$is the saturation vapor pressure at ambient temperature $(kPa)$, and $g_{v}$ is the total water vapor conductance per surface leaf area $(molm^{-2} s^{-1})$.

$C_{i}$ is obtained using **equation S8**.

$C_{i}=C_{a}-A\left( \frac{1.6}{g_{s}}+\frac{1.37}{g_{b}} \right)P_{a}$ (S8)

$C_{a}$ is the ambient CO_2_ partial pressure $(\mu bar)$, $g_{b}$ is the boundary layer conductance to water vapor $(molm^{-2} s^{-1})$.

Transpiration (E) is estimated using **equation (S9).**

$E=2g_{v}\left[ \frac{e_{s} \left( T_{L} \right)- e_{a}}{P_{a}} \right]$ (S9)

Where, $e_{s}$ is the vapor pressure at the leaf surface $(kPa)$, $e_{a}$ is the vapor pressure in the ambient air $(kPa)$, $P_{a}$ is the atmospheric pressure $(kPa)$, $T_{L}$ is the leaf temperature $(℃)$, $g_{v}$ is the total water vapor conductance per surface leaf area $(molm^{-2} s^{-1})$ estimated using **equation (S10)**.

$g_{v}=0.5\frac{g_{s}g_{b}}{g_{s}+g_{b}}$ (S10)

$g_{s}$ is the stomatal conductance to water vapor (**equation (S5)**) and $g_{b}$ , boundary layer conductance to water vapor $(molm^{-2} s^{-1})$ is calculated using **equation (S11)**

$g_{b}=0.147\sqrt{\frac{u}{d}}$ (S11)

Where $u$ is the wind speed, and $d$ is the leaf dimension.

All four sets of equations (**equations (S1-S4, S5-S6, S7-S8, and S9-S11)**) are interrelated. FvCB model requires the value of $C_{i}$ and $T_{L}$ along with other variables. The stomatal conductance model requires net photosynthesis from the FvCB equation (**equation (S1)**). The $C_{i}$is a function of both A and $g_{s}$ (**equation (S8)**). T_L_ is determined from the energy balance equation by using air temperature, heat conductance, and water vapor conductance (**equation (S7)**). These equations are solved iteratively using the Newton-Raphson method as discussed in Kim and Lieth^5^ and Yang et al.^4^. Transpiration and gross canopy photosynthesis are estimated from the leaf level response scaled up to the whole canopy using the sunlit/shaded leaf area fractions ^6^. The same approach is already incorporated in plant models for rose^5^, rice^7^, maize^4^, and potato^8^. The results indicated that the models could capture the growth and development responses in varying soil-plant-atmosphere conditions ^4,8,9^. The process marked in the green box (PNET and TRANSP) in **Fig. 2** is replaced by the Gas exchange model.

Table S1. Gas exchange model parameters ^5,10,11^. These parameters are used in case studies 1 and 2.

| Parameters | Definition | Unit | Values |
| --- | --- | --- | --- |
| V_cm25_ | Photosynthetic rubisco capacity at 25ºC | μmol e^-1^ m^-2^ s^-1^ | 140 |
| J_m25_ | Potential rate of electron transport at 25^º^C | μmol m^-2^ s^-1^ | 225 |
| TPU_25_ | Rate of triose phosphate utilization at 25ºC | μmol m^-2^ s^-1^ | 15 |
| R_d25_ | Mitochondrial respiration in the light at 25ºC | μmol m^-2^ s^-1^ | 1 |
| K_c25_ | Michaelis Menten constant of Rubisco for CO_2_ | μbar | 404.9 |
| K_o25_ | Michaelis Menten constant of Rubisco for O_2_ | μbar | 278.4 |

**References**

1. Farquhar, G. D., von Caemmerer, S. & Berry, J. A. A biochemical model of photosynthetic CO2 assimilation in leaves of C3 species. *Planta* **149**, 78–90 (1980).

2. Ball, J. T., Woodrow, I. E. & Berry, J. A. A model predicting stomatal conductance and its contribution to the control of photosynthesis under different environmental conditions. in *Progress in photosynthesis research* 221–224 (Springer, 1987).

3. Tuzet, A., Perrier, A. & Leuning, R. A coupled model of stomatal conductance, photosynthesis and transpiration. *Plant, Cell & Environment* **26**, 1097–1116 (2003).

4. Yang *et al.* Simulating Canopy Transpiration and Photosynthesis of Corn Plants under Contrasting Water Regimes Using a Coupled Model. *Transactions of the ASABE* **52**, 1011–1024 (2009).

5. Kim & Lieth, J. H. A Coupled Model of Photosynthesis, Stomatal Conductance and Transpiration for a Rose Leaf (Rosa hybrida L.). *Annals of Botany* **91**, 771–781 (2003).

6. De Pury, D. G. G. & Farquhar, G. D. Simple scaling of photosynthesis from leaves to canopies without the errors of big-leaf models. *Plant Cell Environ* **20**, 537–557 (1997).

7. Li, S. *et al.* Application of a coupled model of photosynthesis, stomatal conductance and transpiration for rice leaves and canopy. *Computers and Electronics in Agriculture* **182**, 106047 (2021).

8. Fleisher, D. H., Timlin, D. J., Yang, Y. & Reddy, V. R. Simulation of potato gas exchange rates using SPUDSIM. *Agricultural and forest meteorology* **150**, 432–442 (2010).

9. Kim *et al.* Modeling Temperature Responses of Leaf Growth, Development, and Biomass in Maize with MAIZSIM. *Agronomy Journal* **104**, 1523–1537 (2012).

10. Baker, J. T. *et al.* Canopy Gas Exchange Measurements of Cotton in an Open System. *Agron. J.* **101**, 52–59 (2009).

11. Medlyn, B. E. *et al.* Temperature response of parameters of a biochemically based model of photosynthesis. II. A review of experimental data. *Plant, Cell & Environment* **25**, 1167–1179 (2002).
